# Task allocation and site fidelity jointly influence foraging regulation in honeybee colonies

**DOI:** 10.1098/rsos.170344

**Published:** 2017-08-30

**Authors:** Thiago Mosqueiro, Chelsea Cook, Ramon Huerta, Jürgen Gadau, Brian Smith, Noa Pinter-Wollman

**Affiliations:** 1 BioCircuits Institute, University of California San Diego, La Jolla, CA, USA; 2 School of Life Sciences, Arizona State University, Tempe, AZ, USA; 3 Department of Ecology and Evolutionary Biology, University of California Los Angeles, Los Angeles, CA, USA; 4 Institute for Evolution and Biodiversity, University of Münster, Münster, Germany

**Keywords:** *Apis mellifera*, collective behaviour, exploitation, exploration, group composition, persistence

## Abstract

Variation in behaviour among group members often impacts collective outcomes. Individuals may vary both in the task that they perform and in the persistence with which they perform each task. Although both the distribution of individuals among tasks and differences among individuals in behavioural persistence can each impact collective behaviour, we do not know if and how they jointly affect collective outcomes. Here, we use a detailed computational model to examine the joint impact of colony-level distribution among tasks and behavioural persistence of individuals, specifically their fidelity to particular resource sites, on the collective trade-off between exploring for new resources and exploiting familiar ones. We developed an agent-based model of foraging honeybees, parametrized by data from five colonies, in which we simulated scouts, who search the environment for new resources, and individuals who are recruited by the scouts to the newly found resources, i.e. recruits. We varied the persistence of returning to a particular food source of both scouts and recruits and found that, for each value of persistence, there is a different optimal ratio of scouts to recruits that maximizes resource collection by the colony. Furthermore, changes to the persistence of scouts induced opposite effects from changes to the persistence of recruits on the collective foraging of the colony. The proportion of scouts that resulted in the most resources collected by the colony decreased as the persistence of recruits increased. However, this optimal proportion of scouts increased as the persistence of scouts increased. Thus, behavioural persistence and task participation can interact to impact a colony's collective behaviour in orthogonal directions. Our work provides new insights and generates new hypotheses into how variations in behaviour at both the individual and colony levels jointly impact the trade-off between exploring for new resources and exploiting familiar ones.

## 1. Introduction

Group composition impacts the emergence of collective behaviours. Individuals that comprise a group vary both in which tasks they perform [1,2] and in how persistently they perform them, i.e. how many times they repeatedly perform a task [3,4]. The effect of allocation of workers to different tasks on the collective behaviour of colonies has been studied extensively [5] with the underlying assumption that dividing the labour among group members will increase the overall efficiency of the group, as it does in human industrial societies [6]. However, variation among individuals in how persistently they perform a task is striking. This behavioural variation can undermine the efficiency that is often associated with task specialization [7,8] because individuals that are not persistent either do not perform a large proportion of the task or incur the costs of task switching [9,10]. Although recent work has begun to examine the impact of variation in individual persistence in performing a particular task on collective behaviours [11,12], we do not know how task allocation and variation in persistence interact to impact collective outcomes.

Behavioural persistence has now been documented extensively throughout the animal kingdom [3] including in social insects [4]. Some ant workers are more persistent in performing a certain task than others [13], and honeybee workers vary in how persistently active they are [14,15]. Behavioural persistence can impact how individuals in a group interact with one another and therefore affect the collective behaviours that emerge from these interactions [12,16]. A growing understanding of the mechanisms that underlie behavioural persistence is paving a path for understanding how variation in behavioural persistence affects collective outcomes. For example, the decision of a honeybee to leave the hive and start foraging is influenced by the bee's genome [17–22]. Furthermore, genetic variation underlies individual differences in learning abilities, which might influence the likelihood of a bee to make certain types of foraging decisions, such as staying at a resource patch [23–27].

Honeybees exhibit variation in foraging behaviours at both the worker and colony levels [28–30]. Understanding the mechanisms that underlie honeybee foraging decisions is especially important because of their economic importance for honey production and crop pollination [31,32]. Consistent behavioural variation across workers within honeybee colonies has potential fitness consequences [29]. Although the regulation of foraging behaviour in honeybees has been studied for a long time [33], and much is known, for example, about how foragers respond to resource availability [21,34], we still do not know what mechanisms may underlie variation among colonies in collective foraging.

Many tasks in honeybee colonies are related to foraging. For example, some foragers collect pollen, while others specialize in collecting nectar [33,35], and an animal's genotype influences a bias for one or the other [36–38]. Nectar foragers further vary in their propensity to leave the nest to find new food. Experienced foragers that spontaneously leave the hive to explore the environment are referred to as ‘scouts’ or ‘primary searchers’ [39–42]. When these scouts return to the hive, they recruit other foragers to the food patches they found, and these bees are referred to as ‘recruits’. Scouts communicate to recruits the direction, distance and quality of newly found resources using the waggle dance [33,43], thus reducing waste of energy spent when searching for food over both long and short timescales [44,45], and dangers, such as predation [34,46,47]. Although exploration of the environment for new food sources is a task exclusive to scouts, they can contribute to the exploitation of resources, alongside the recruits, through repeated visits to the same source [21,48]. We define persistence of a forager as the average number of repeated visits it performs to each particular resource, regardless of whether it is a scout or a recruit. Thus, both scouts and recruits with lower persistence can contribute to a colony's exploration of the environment because low-persistence scouts will travel to different resources and low-persistence recruits will stop foraging quickly and become available to be recruited to new locations. High persistence of both scouts and recruits can contribute to the colony's exploitation of resources through repeated visits to a profitable source but can also hinder the efficiency of collective foraging if other, more profitable resources are available. Honeybees choose between exploring for new resources or exploiting familiar ones based on colony [41] and individual information [49,50]. Thus, the trade-off between exploration and exploitation can be adjusted both at the colony level, through allocation of foragers to either scouts or recruits, and at the individual level, through variation in the persistence of visits to a known food source.

Although the trade-off between exploration and exploitation has been previously examined in honeybees by addressing the differences between scouts and recruits [21,51], the role of behavioural persistence in visiting the same resource, i.e. site fidelity, has thus far been overlooked. Because foraging is energetically costly [49,52], greater persistence does not always translate into greater efficiency. To examine the joint role of task allocation and behavioural persistence in the regulation of foraging by honeybees, we considered how the ratio between scouts and recruits and the persistence of returning to a particular resource jointly affect the collective resource acquisition by a colony. Specifically, we examine how behavioural persistence of (i) the entire colony, (ii) scouts or (iii) recruits affects collective foraging when different proportions of foragers are allocated either to scouting or to being recruited. Our findings provide new and realistic insights on how behavioural variation at more than one level of organization impacts collective outcomes.

## 2. Material and methods

### 2.1. Agent-based model

To examine the joint impact of task allocation and behavioural persistence on collective behaviour, we developed a spatially explicit agent-based model. Simulated honeybees foraged in an open, continuous, two-dimensional space. The hive was set at the origin of the space and three unlimited resource patches were uniformly distributed around it at a fixed distance of 15 m from the hive, with equal distances between neighbouring sites. We simulated two types of foragers, scouts and recruits, which varied in their flight patterns as detailed below. To determine the effects of behavioural persistence on colony outcomes, we examined the proportion of scouts that leads to the maximum amount of resources collected by the colony under different regimes of behavioural persistence. A description using the Overview, Design concepts and Details (ODD) protocol [53] and the source code of our model can be found on Github [54]. In the following sections, we define the flight dynamics of foragers (section ‘Flight dynamics’) and describe the variables used to quantify colony success (section ‘Collective outcomes’) within the agent-based model.

### 2.2. Flight dynamics

Flight dynamics of all foragers were modelled as a random walk with drift [55,56]. At the beginning of each simulation (*t* = 0), the position of each bee *i* was ***x****_i_*(0) = (0,0), i.e. all bees were at the hive. Each bee was assigned a different drifting vector ***v****_i_*, which determined its flight direction when leaving the hive, and its flight pattern is described as

2.1 $$\text{d}x_{i}(t)=v_{i}\;\text{d}t+\sigma_{i}\;\text{d}W_{t},$$

where $\sigma_{i}\;\text{d}W_{t}$ is the random contribution to the distance and angle a bee moved. This term has a normal distribution with a mean of zero and variance of *σ_i_*, thus closely resembling a diffusion process [57]. Specifically, 1/*σ_i_* measures the precision of the flight. Because *E*[d***W****_t_*] = 0, the average velocity of the *i*th bee was ***v****_i_*, and its magnitude $v_{i}=|v_{i}|$ defined the average flight velocity. The stochastic dynamics in equation (2.1) produce slight variation among bees in their flight patterns to avoid an unrealistic scenario in which bees take a straight line between two points. Using the Euler–Maruyama method [58], equation (2.1) can be solved numerically using

**Figure 1.**
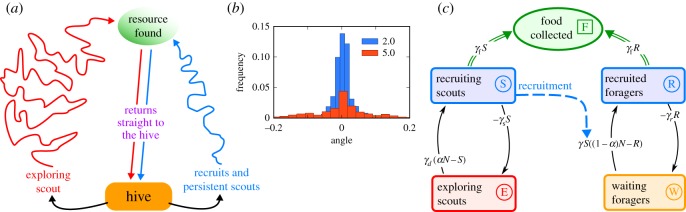
Flight dynamics of scouts and recruits. (*a*) Scouts left the hive at the beginning of the simulation and once they found a resource, they recruited other foragers, referred to as ‘recruits’. (*b*) Variance of the scouts' deviations from a straight path on outgoing trips ($\tilde{\sigma }=5$, red) was larger than that of the recruits and persistent scouts ($\tilde{\sigma }=2$, blue), resulting in greater spatial dispersion. (*c*) System dynamics approach based on a compartmental model, with square boxes representing the states of foragers and the green circle representing the amount of food retrieved by all foragers. Black arrows are state-transition rates (see equations (2.6) and (2.7)); the blue dashed arrow represents the recruitment of foragers by scouts; the green double arrows represent foragers delivering food to the hive.

2.2 $$x_{i}(t+\Delta t)=v_{i}\Delta t+\sqrt{\Delta t}\sigma_{i}W_{t}+x_{i}(t)=v_{i}\Delta t+{\tilde{\sigma }}_{i}W_{t}+x_{i}(t),$$

with Δ*t* being a fixed time step, and ${\tilde{\sigma }}_{i}=\sqrt{\Delta t}\sigma_{i}$. At the beginning of each simulation (*t* = 0), all scouts left the hive, with drifting vectors *v_i_* assigned from a uniform distribution, and continued flying until they found a resource. Once a scout detected a resource, it returned to the hive to recruit other foragers, referred to as ‘recruits’. Scouts and recruits differed in the precision of their flight: ${\tilde{\sigma }}_{i}$ of scouts was larger (${\tilde{\sigma }}_{i}=5$) than that of recruits, resulting in flight paths that covered a larger area than recruits (figure 1). The dispersion of recruited bees (${\tilde{\sigma }}_{i}=2$) was fitted using data from experiments with feeders positioned at distances varying from metres to kilometres [33]. To differentiate between the flight patterns of bees that are exploring the environment and those that are exploiting a resource patch, are familiar with their location, and are therefore faster and more precise, we assigned *v_i_* = 1 to scouts and *v_i_* = 1.5 to recruits, following [59]. Foragers that reached the limit of the simulated area were set back to the hive instantly to start foraging again.

During recruitment, scouts communicated the location and distance of the newly found resource. The recruiting scout remained at the hive for 1 min (approx. 50 time steps in the numeric simulations) to simulate the time it would take to recruit foragers using the waggle dance [33]. During this period, an average of five randomly selected recruits left the hive in the direction of the resource. Recruiting on average 1, 5 or 10 foragers by each scout did not qualitatively change the results of our simulations. For simplicity, only the recruitment by scouts is considered here, and we examine the effect of adding recruitment by recruits in the electronic supplementary material, figure S1. Distance and quality of food patches are also communicated in the waggle dance [33,60], and variation in distance and quality could be easily incorporated in further investigations of our model by varying the number of recruits that respond to each recruiting forager and the time that each scout spends recruiting.

Each of the newly recruited bees left the hive with their drifting vectors pointing exactly towards the location reported by the recruiting scouts, analogous to previous experiments [61]. The direction of this drifting vector is the deterministic portion of the flight dynamics (see ***v****_i_*d*t* in equation (2.1)), which is accompanied by a stochastic contribution from $\sigma_{i}\;\text{d}W_{t}$. Recruited bees exploited the first resource they found during their trips. The dispersion of recruited bees (*σ* = 2) was fitted using data from experiments with feeders [32]. Because the stochastic element of the flight of a recruited bee is very small compared with the size of the resource patches in our simulations, bees always exploited the same resource patch that was reported to them. The effect of communicating the distance to the source was modelled by slightly changing the dynamics in equation (2.1) to

2.3 $$\text{d}x_{i}(t)=v_{i}\alpha (|x_{r}-x_{i}(t)|)\;\text{d}t+\sigma_{i}\;\text{d}W_{t},$$

where *α*(*x*) is any function that goes to zero when *x *→ 0 and ***x***_r_ is the location of the resource reported. This turned the flight dynamics into a purely random walk (i.e. without bias) near the location of the reported resource. For simplicity, we used a Heaviside function that removed all bias in the flight dynamics when the forager was less than 2 m from the resource:

2.4 $$\alpha (x_{r}-x_{i}(t))=\left\{ \begin{array}{ll} 1, & \text{if }|x_{r}-x_{i}(t)|-2;\\ 0, & \text{otherwise}. \end{array}\right.$$

During our simulations, scouts and recruits obtained resources for the colony. Upon obtaining a resource, foragers (both scouts and recruits) returned to the hive in a straight line, with constant velocity *v_i_*, carrying one resource unit, equivalent to 1.0 ± 0.3 µl [33]. If a forager reached the boundaries of the area considered in the simulation, it was reassigned to the hive, without bringing food, to begin foraging again. For simplicity, this reassignment was instantaneous, but adding a return trip or changing the distance explored by these foragers before they return to the hive did not change our findings (electronic supplementary material, figure S2).

Each forager, scout or recruit, was assigned a persistence value *π_i_*, defined as the number of consecutive trips it performed to each resource location. If the persistence of a scout was greater than 1, its *v_i_* and *σ_i_* after the first trip were set to those of recruits and its flight dynamics was adjusted to follow equation (2.2). Scouts that completed *π_i_* trips to the same location randomly changed their drifting vector and began scouting again. Recruits that completed *π_i_* trips remained at the hive until they were recruited again.

### 2.3. Collective outcomes

To examine the impact of colony composition on collective foraging success, we simulated colonies with different ratios between scouts and recruits. For simplicity, we neglect the effect of inactive foragers [42] and we fixed the number of scouts and recruits during each simulation. Simulated colonies consisted of 300 foragers that were allowed to forage for 7 h in an area equivalent to 36 × 36 m = 1296 m^2^. These values were selected based on empirical data on honeybee foraging [33]. Because each simulation reflected just one day of foraging, we assumed that resources were never depleted during a simulation and that the ratio between scouts and recruits was fixed.

The colony-level outcome was measured as the total amount of resources retrieved by all the bees in the colony. For each simulation *j* that we ran, we recorded the resources *f_j_*(*t*) collected over time. Because of the stochastic nature of our simulations, the amount of resources collected at each time point over all our *n* simulations followed a bell-shaped distribution with a variance *V*. To ensure that all conditions tested (i.e. proportion of scouts and various persistence values) produced the same 90% confidence interval *w* for the estimation of the average amount of resource collected (see shaded area in figure 3*a,b*), we used the central limit theorem to set the number of simulation runs to *n* = 4*V*^2^/*w*. Because the mean of the total amount of resources collected was of the order of thousands of microlitres, we set *w* = 50 µl, resulting in *n* of approximately 120. We estimated the average amount of resources collected at every time point in all *n* simulation runs as *f*(*t*) = *E*[*f_j_*(*t*)] (see lines in figure 3*a,b*).

### 2.4. System dynamics model

To complement our understanding of how behavioural persistence and recruitment by scouts in the agent-based model combine to result in complex outcomes, we used a coarse-grained formalism based on ordinary differential equations that describe the system's dynamics (figure 1*c*), similar to [62]. We consider the following dynamical variables: *E*(*t*), the number of scouts exploring the environment; *S*(*t*), the number of scouts that are bringing food back to the hive; *R*(*t*), activated recruits; and *W*(*t*), potential recruits waiting inside the hive. Let *N* be the number of foragers in the colony; then *αN* is the total number of scouts and (1 − *α*)*N* is the total number of recruits. Thus, *E*(*t*) = *αN *− *S*(*t*) and *W*(*t*) = (1 − *α*)*N *− *R*(*t*). Because *S*(*t*) and *R*(*t*) represent the total number of foragers collecting food at any given time, we refer to them as *active foragers*. If we define *γ* as the rate at which active scouts *S*(*t*) recruit inactive recruits *W*(*t*), then the increase in the number of active recruits is described by γSW=γS((1−α)N−R). A simple model describing the rate of change in number of scouts and number of recruits can be defined by two differential equations:

2.5*a* $$\frac{\text{d}S}{\text{d}t}=\gamma_{d}(\alpha N-S)-\gamma_{s}S$$

and

2.5*b* $$\frac{\text{d}R}{\text{d}t}=\gamma S((1-\alpha )N-R)-\gamma_{r}R,$$

where *γ*_d_ is the rate at which scouts find a new resource and start exploiting it; *γ*_s_ is the rate at which these scouts stop collecting food and resume exploring for new resources; and *γ*_r_ is the rate at which the recruited foragers stop collecting food and begin waiting to be recruited again. Finally, the cumulative amount of food collected by active foragers *F*(*t*) can be formulated as

2.6 $$\frac{\text{d}F}{\text{d}t}=\gamma_{f}(S+R),$$

with *γ*_f_ being the rate at which bees collect food while exploiting a particular resource.

In this compartmental model, behavioural persistence, in the form of repeated visits to a particular resource site, is defined according to the rates at which foragers stop exploiting particular resources. Both 1/*γ*_s_ and 1/*γ*_r_ represent the characteristic durations of exploiting a particular resource by scouts or recruits. Dividing these characteristic durations by the average time interval ⟨Δ⟩ between each visit to the feeder (which was experimentally evaluated as described in section ‘Behavioural experiments and parameter estimation’) gives the average number of visits to one resource. Thus, to link the rates at which foragers stop exploiting a particular resource with the persistence parameter in the agent-based model, we define

2.7*a* $$\gamma_{s}=\frac{{\bar{\gamma }}_{s}}{\pi^{s}\langle \Delta \rangle }$$

and

2.7*b* $$\gamma_{r}=\frac{{\bar{\gamma }}_{r}}{\pi^{r}\langle \Delta \rangle }.$$

Defining the relationship between *γ*_s_ and *γ*_r_ and persistence, as simulated in the agent-based model, allows us to analyse the compartmental model without having to fit a different value of *γ*_s,r_ for each *π*^s,r^, reducing the complexity of our compartmental model. The parameters ${\bar{\gamma }}_{s}$, ${\bar{\gamma }}_{r}$, *γ*_f_ and *γ*_d_ were fitted using simulation data from the agent-based model.

### 2.5. Behavioural experiments and parameter estimation

To assess persistence empirically, we observed the visitation of 323 honeybee (*Apis mellifera* L.) foragers from five different colonies during the winter (between 3 and 26 February 2016). Each colony was tested on a different day and was presented with two feeders, each containing 1 M sucrose solution on which the foragers fed ad libitum. We trained bees to find feeders, positioned at 3, 5 or 10 m from the hive, 1 day before the experiments began, following [18] and comparable to other studies that examine 20 m [63]. These resources were never depleted despite their proximity to the hive. During the time of our experiments, there were few naturally blooming plants and our feeders were very attractive to the bees. We marked workers for individual identification using water-based acrylic paint markers (Montana) and recorded the time at which each bee visited a feeder using the software EventLog [64]. We recorded 1307 trips. Work with invertebrates does not require ethics committee approval and all fieldwork was conducted on university property. All collected data are publicly available [65].

**Figure 2.**
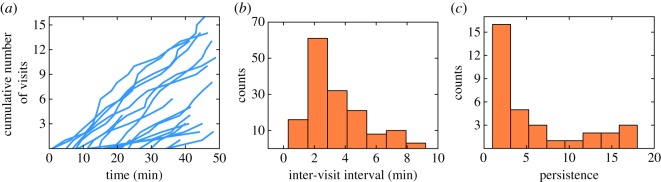
Empirical results of 206 foraging trips performed on one day by 33 different honeybee foragers from one representative colony of the five colonies we tested. The feeder was positioned 5 m from the hive. (*a*) Number of visits over time. Each line represents one bee and *t* = 0 reflects the first bee's first visit to the feeder. (*b*) Distribution of intervals between consecutive visits to a single feeder. (*c*) Distribution of persistence, i.e. the number of return visits by each bee to one of two feeders. The average persistence was 6.1 ± 0.3.

We estimated the values for the parameters in our model based on the empirical observations. Interestingly, all bees exhibited the same rate of visits to the feeders (figure 2*a*), which was 0.4 ± 0.2 visits per minute (figure 2*b*). This visitation rate allowed us to set the model parameter ***v***_i_ for flight velocity to a constant value for all foragers after their first visit at a resource. The empirical distribution of intervals between consecutive visits to the feeder (figure 2*b*) informed the visitation interval of our model. The observed average visitation interval ⟨t⟩ was linearly related to the distance *d* between the hive and the feeder (*R* = 95%, electronic supplementary material, figure S3): ⟨Δ⟩=αd+β, with *α* = 2.3 ± 0.3 and *β* = 0.28 ± 0.05. Finally, the observed distribution of persistence was geometric or negative binomial (figure 2*c*), with an average of ±90% CI = 6.1 ± 0.3. This means that making the decision to stop exploiting a particular patch had a probability of 16%, based on the value of the lambda parameter of a geometric distribution that was fitted to the data. Because the largest number of observed return visits by a single bee was 22, we restricted our persistence parameter *π_i_* to range between 1 and 30.

## 3. Results

**Figure 3.**
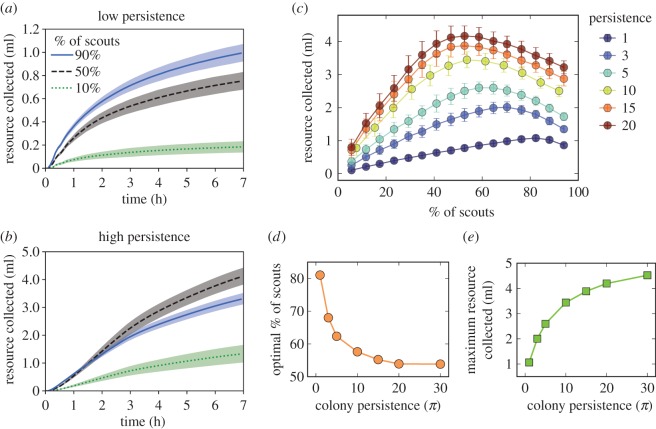
The relationship between colony persistence *π* and the proportion of scouts affects the amount of resources collected by a colony. The amount of resources collected over time by a simulated colony in which all foragers have either (*a*) low persistence (*π* = 1) or (*b*) high persistence (*π* = 20) for three different proportions of scouts. Shaded areas represent 1.5 s.d. (*c*) Total amount of resource collected throughout the entire simulation as a function of the proportion of scouts in the colony for different values of persistence of all foragers (*π*). Bars are the standard deviation across all simulation runs. (*d*) Optimal proportion of scouts plateaus near 50% as *π* increases. Points are the results from our agent-based model and the line is the result from the systems dynamics approach (equation (3.4)). (*e*) Maximum amount of resources collected scales sublinearly with *π*. Points are the results from our agent-based model, and the line is the result from the systems dynamics approach (equation (3.3)).

The proportion of scouts that maximized the amount of resources a colony collected by the end of the simulation, referred to as the ‘optimal proportion of scouts’, changed with the persistence of visiting a resource. The amount of resources collected in all simulations increased over time (figure 3*a,b*). The total amount of resources collected at the end of the simulation was different among the various proportions of scouts. When both scouts and recruits lacked persistence, i.e. each bee made only a single trip to the feeder (*π* = 1), more resources were collected as the proportion of scouts increased (figure 3*a*). However, as the persistence of all foragers increased from *π* = 1 to 20, a greater proportion of scouts in a colony did not necessarily result in more resources collected. For example, when persistence was set at *π* = 20, colonies with 50% scouts outperformed colonies with 90% scouts (figure 3*b,c*). For each persistence value *π,* we found the optimal proportion of scouts, i.e. the proportion of scouts that resulted in the most resources collected by the end of the simulation (after 7 h of foraging; figure 3*c*). This optimal proportion of scouts decreased with persistence and saturated after *π* > 20 (figure 3*d*). However, the absolute amount of resources collected per colony continued to grow when persistence increased beyond 20 visits per individual (*π* > 20; figure 3*e*). Changing the number of resource patches impacted the total amount of resources collected by the colony, but the optimal proportion of scouts still decreased with the persistence of the colony (electronic supplementary material, figure S4). This result led us to further investigate the relationship between the total amount of resources collected and the persistence of recruits and of scouts, as detailed below.

The system dynamics model allows us to further evaluate the processes that determine the optimal proportion of scouts using the stable solutions for scouts (*S*) and recruits (*R*),

3.1*a* $$S^{*}=S(\infty )=\alpha N\left(\frac{\gamma_{d}}{\gamma_{d}+\gamma_{s}}\right)$$

and

3.1*b* $$R^{*}=R(\infty )=(1-\alpha )N\left(\frac{1}{1+\gamma_{r}(\gamma_{d}+\gamma_{s})/\gamma \gamma_{d}\alpha N}\right).$$

The expressions inside the parenthesis in equations (3.1*a*) and (3.1*b*) represent, respectively, the proportions of scouts and recruits that become active after a long time, i.e. asymptotically. These solutions reveal that the optimal proportion of active scouts is determined solely by the ratio between the rate at which scouts discover new resource sites, *γ*_d_, and the rate at which they abandon them, *γ*_s_. For a fixed rate of discovery, *γ*_d_, the number of active scouts increases almost linearly with the persistence of scouts, saturating for large values of persistence, i.e. when *γ*_s_ → 0. However, the number of active recruits does not depend directly on the persistence of scouts, but on the number of scouts, α*N*, and the rate of recruitment, γ. From equation (2.7), the amount of food collected, *F*(*t*), grows asymptotically at a fixed rate,

3.2 $$\frac{\text{d}F}{\text{d}t}=\gamma_{f}(S^{*}+R^{*})=\gamma_{f}N\chi_{2}\alpha \left[1+\chi_{1}\frac{1-\alpha }{1+\alpha \chi_{1}\chi_{2}}\right],$$

where $\chi_{1}=\gamma N/\gamma_{r}$ measures the trade-off between recruitment and the persistence of recruits; and $\chi_{2}=\gamma_{d}/(\gamma_{d}+\gamma_{s})$ is the ratio between the rate of discovering new resource sites *γ*_d_ and the rate of abandoning a site *γ*_s_ (same expression as in (3.1*a*)). If there are no scouts, *α* = 0, then no food is collected, which agrees with the agent-based model (figure 3*c*). Because the rate *γ*_d_ at which new resources are discovered is constant in our model, the amount of food collected, *F*(*t*), always grows and does not present a stable solution. However, the asymptotic speed at which *F*(*t*) grows, shown in equation (3.2), changes with the proportion of scouts in the colony, *α*. Thus, for long times, the amount of food collected, *F*(*t*), grows linearly, and comparing the rate of increase among different persistence values is equivalent to comparing the relative values of *F*(*t*) at a fixed time point *t*, as in figure 3*c,d*. To simplify the dependence of the rate of increase of *F*(*t*) on the proportion of scouts, *α*, in equation (3.2), we use the Taylor expansion up to second order in *α*:

3.3 $$\gamma_{f}(S^{*}+R^{*})=\gamma_{f}N\chi_{2}[\alpha (1+\chi_{1})-\alpha^{2}\chi_{1}(\chi_{1}\chi_{2}+1)]+O(\alpha^{3}),$$

with O being the ‘big O’ notation, i.e. it refers to the remaining terms that are polynomials in *α* of order 3 or higher, and have a small contribution to equation (3.3) because 0 < *α* ≤ 1. Thus, the asymptotic rate of resource collection is a concave function whose maximum depends on *α*, the proportion of scouts in the colony, in accordance with the results from our spatially explicit agent-based model (figure 3*c*). The optimal proportions of scouts predicted by the system dynamics agree perfectly with the results of the agent-based model (lines in figure 3*c,d*). However, the curvature of the amount of resources collected in relation to the proportion of scouts slightly differs between the system dynamics and the agent-based models (electronic supplementary material, figure S5).

**Figure 4.**
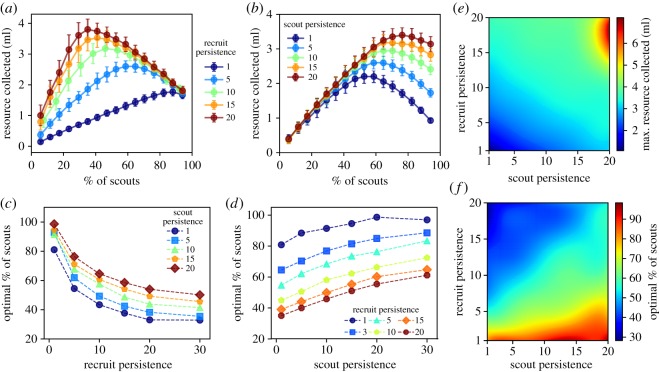
Differences in collective foraging due to the persistence of either scouts (*π*^s^) or recruits (*π*^r^ in the agent-based model). Total amount of resources collected by a colony as a function of the proportion of scouts when (*a*) the persistence of scouts is set to *π*^s^ *=* 5 for the following values of persistence of recruits: *π*^r^ = 1,5,10,15,20 and (*b*) the persistence of recruits is set to *π*^r^ = 5 for the following values of persistence of scouts: *π*^s^ = 1,5,10,15,20. Bars are the standard deviation across all simulation runs. Proportion of scouts that resulted in maximal amount of resource collected as a function of (*c*) recruit persistence for different values of fixed scout persistence *π*^s^ and (*d*) scout persistence for different values of fixed recruit persistence *π*^r^. (*e*) Heat map of the maximum amount of resources collected for different values of scout *π*^s^ and recruit *π*^r^ persistence jointly. (*f*) Heat map of the proportion of scouts that led to the maximum amount of resources collected for different values of scout *π*^s^ and recruit *π*^r^ persistence jointly.

**Figure 5.**
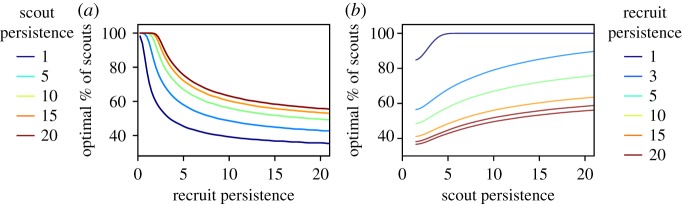
The systems dynamics approach captures the opposing effects of scout and recruit persistence on the optimal proportion of scouts. (*a*) Change in optimal per cent of scouts due to change in the persistence of scouts. (*b*) Change in optimal per cent of scouts due to change in the persistence of recruits.

**Figure 6.**
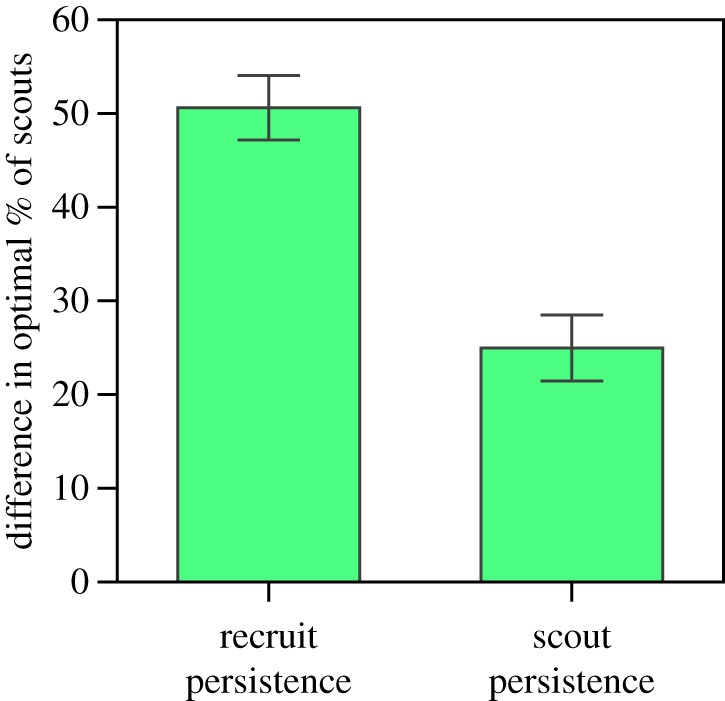
The effect of increase in recruit persistence on the proportion of scouts that resulted in an optimal amount of resource collected was double that of the effect of increase in scout persistence. Bars are the standard deviation across all persistence values.

Changes in the persistence of scouts had the opposite effect from changes in the persistence of recruits on the proportion of scouts that maximized collective resource collection. In the agent-based model, while the optimal proportion of scouts decreased with the persistence of recruits *π*^r^ (figure 4*a*), this proportion increased with the persistence of scouts *π*^s^ (figure 4*b*). This opposite dependence of the optimal proportion of scouts on *π*^s^ and *π*^r^ was observed for a wide range of both scout and recruit persistence values (figure 4*c,d*). Our system dynamics model also reproduces this dependence (see lines in figure 5). The combined scout–recruit persistence with the best collective outcome, i.e. greatest amount of resources collected, resulted from the largest persistence values of both scouts and recruits (figure 4*e*) when approximately 60% of the foragers were scouts (figure 4*f*). The opposing dependence of the optimal proportion of scouts on scout and recruit persistence is captured by our system dynamics (figure 5), through the relationship between *γ*_s_ and *γ*_r_ in equation (3.3). Interestingly, changes in the persistence of recruits resulted in a 50% change in the optimal proportion of scouts, whereas changes in the persistence of scouts resulted in only a 25% change in this proportion (figure 6).

## 4. Discussion

Social groups constantly adjust their collective behaviour to changes in their surroundings. However, an understanding of how these adjustments emerge is still scant. Our models show that both colony-level composition, i.e. the ratio between scouts and recruits, and individual-level traits, such as the persistence of foragers, interact to impact collective foraging. We found that the balance between the proportion of bees scouting and behavioural persistence allows a colony to acquire more resources and allocate fewer individuals to the potentially costly activity of scouting. Scouts may expend considerable energy flying around in search for new resources, and they can be preyed upon or potentially lose their way home [34]. In our simulations, colonies with high persistence, *π* = 20, collected almost five times more resources than those with low persistence, *π* = 1 (figure 3*c*). The trade-off between exploring for new resources and exploiting known ones resulted in a different optimal proportion of scouts for each value of persistence (figure 3). As persistence increased, the proportion of scouts required for collecting the maximal amount of resources decreased to a minimum near 50% (figure 3*b,c*), because exploiting known resources required fewer scouts to find new resources. Previous studies estimated that the percentage of scouts in honeybee colonies is between 5 and 35% [39]. These numbers are slightly lower than the optimal proportions we found in our simulations. This difference between empirical and simulated results can be eliminated by increasing the number of bees that respond to a waggle dance in our simulations (electronic supplementary material, figure S6) and without changing any other parameter in the model, or affecting any of our conclusions regarding persistence and task allocation (compare figure 3 with electronic supplementary material, figure S6). Changing the number of foragers (from 100 to 1500) did not qualitatively change how persistence and colony composition interacted to achieve optimal resource collection (electronic supplementary material, figures S7 and S8), although, in agreement with previous modelling efforts [59], larger colonies did induce faster collection of resources. Lastly, the effect of including recruitment by recruits on the optimal proportion of scouts was the same as that of increasing the number of recruited foragers by scouts per waggle dance (electronic supplementary material, figure S1).

Changing the persistence of scouts had a different impact on collective foraging from changing the persistence of recruits. We found that an increase in the persistence of recruits resulted in a decrease in the proportion of scouts required for collecting the maximal amount of resources. By contrast, an increase in the persistence of scouts resulted in an increase in the proportion of scouts required for collecting the maximal amount of resources (figure 4*a–d*). This result suggests that the persistence of recruits was the predominant factor impacting the optimal proportion of scouts when varying the persistence of all foragers (figure 3). Indeed, the effect of the persistence of recruits on the proportion of scouts that resulted in an optimal collective outcome had double the impact of persistence of scouts (figure 6). Because recruits spend much time inside the hive, their persistence may change in response to information about the amount of resource stocks in the hive [42,66]. Furthermore, recruits may acquire information from several scouts that are returning from different locations and decide which one to follow and how many trips to make to each location, depending on their relative quality [33,67,68]. If the persistence of recruits is flexible and is determined by integrating information about resources inside and outside the hive, the substantial impact of their persistence on collective foraging that we found suggests that recruits may be the ones driving the adjustment of the colony's exploration–exploitation strategy in response to both external and internal conditions. However, if behavioural persistence is not a flexible trait, perhaps because it is regulated by genetic or epigenetic/developmental factors [17–20,69], our simulations show that a colony can compensate for having highly persistent scouts by allocating more foragers to the scouting task. Interestingly, colonies with comparable persistence for scouts or recruits collected almost the same amount of resources (compare curves with the same colour in figure 4*a,b*), but the optimal proportion of scouts required to achieve the maximal amount of resource collection differed between the two cases. Recent work suggests that persistence can be genetically determined [23], thus one could create colonies with high persistence and examine the proportion of scouting bees emerging in these manipulated colonies. Our model predicts that with high enough persistence, the proportion of scouts should drop by about 40%. Alternatively, because evidence shows that scouting is genetically determined [18], one could also manipulate the proportion of scouts in a colony, and examine if colonies with a greater ratio between the proportion of scouts and persistence gather fewer resources.

Learning the location of a resource did not affect the relationship between persistence and the proportion of scouts. In our simulations, bees communicated the location of newly discovered resources, which is known to increase resource collection in patchy environments [59,60]. Our incorporation of behavioural persistence further enhanced this positive effect of communication by effectively simulating ‘learning’ of the target location. Return flights of scouts to a particular resource became more precise than their initial flight during which they located the resource (figure 1*a*,*b*). Interestingly, when this learning was removed, i.e. flights did not become more precise, the relationship between the optimal number of scouts and persistence was unchanged but the rate of resource collection substantially decreased (electronic supplementary material, figure S9). Thus, when repeatedly returning to the same location does not increase collection efficiency, the total benefits are reduced, but the collective dynamics which dictate the relationship between persistence and optimal proportion of scouts are unchanged. It would be interesting to further investigate the effect of increase in collection efficiency on collective dynamics in primitively social bees that exhibit division of labour but do not share spatial information during recruitment, e.g. bumble bees [44], or halictine bees in which there are no known mechanisms of recruitment [68]. The effects of communication on these dynamics can also be studied in honeybees, for example, by hindering communication through tilting their hive [69], which substantially impairs foraging. Our model predicts that the proportion of scouts that optimizes collection of resources drops by half if recruitment is reduced by a factor of 10 (electronic supplementary material, figure S6). This prediction can be tested by reducing communication in the hive, for example, by turning the hive on its side or capturing recruited bees.

The spatial and temporal abundance of resources can substantially impact foraging behaviour [26,59,60,70]. Indeed, during the development of our model we found that an increase in the number of resource patches caused the total amount of resources collected by the colony to increase for all proportions of scouts, and the optimal proportion of scouts to decrease with the number of patches (electronic supplementary material, figure S4). This finding is consistent with a model of collective foraging in ants [71] which also found that the optimal proportion of scouts is inversely related to the amount of resources in the environment. To examine the relationship between the proportion of scouts in a colony and behavioural persistence, without the confounding effects of resource distribution, we focused only on one spatial setting in our final model. The simulations we present have biological significance for foraging in patchy resources that cannot be depleted in a single day.

In conclusion, we showed that both colony-level composition and individual-level traits interact to impact collective outcomes. The way these levels of organization interact are not affected by the number of resources or colony size (electronic supplementary material). Other complexities, such as the depletion of resources, can be further added to adapt our model to more specific scenarios. Further investigation of the mechanisms that underlie behavioural persistence and task allocation, and examination of the timescales on which these processes act in different species and in different environments will advance our understanding of the collective trade-off between exploration and exploitation. Our model serves as a springboard for such investigations and can be used to generate hypotheses for further empirical work on the regulation of collective behaviour and its response to various environmental conditions.

## Supplementary Material

Supplemental material for Task Allocation and Site Fidelity Jointly Influence Foraging Regulation in Honey Bee Colonies

## Data accessibility

All data collected on behavioural persistence are publicly available on FigShare [65]: https://figshare.com/articles/Task_allocation_and_site_fidelity_jointly_influence_foraging_regulation_in_honey_bee_colonies/3619779. Details and source code of our simulations are publicly available on Github [54]: https://github.com/thmosqueiro/ABBAS.
