# Supplementary material for: Task allocation and site fidelity jointly influence foraging regulation in honeybee colonies
Source: R Soc Open Sci. 2017 Aug 30;4(8):170344. doi: 10.1098/rsos.170344 (PMC5579100; doi:10.1098/rsos.170344)
Supplement: Supplemental material for Task Allocation and Site Fidelity Jointly Influence Foraging Regulation in Honey Bee Colonies [file rsos170344supp1.pdf]

ELECTRONIC SUPPLEMENTARY MATERIAL

Table of contents

Recruitment by recruits....1

Boundary conditions....3

Experiments with feeders at different distances....4

Number of resource patches....5

Comparing Predictions between the System Dynamics and Agent-Based Models....6

Increasing the rate of recruitment....7

Colony size....8

Increasing flight precision in response to finding a resource....9

Recruitment by recruits

Our simulations include only recruitment by scouts, here we note the effects of adding recruitment by recruits to our model. Recruitment by recruits has the same impact on collective foraging as increasing the rate of recruitment by scouts (see above). To avoid instantaneous depletion of available recruits in the hive, the probability that a recruit will recruit new foragers once it returned to the hive ranged from 0 to 0.5. Adding recruitment by recruits does not change the relationship between the proportion of scouts that results in the maximum amount of food collected and the persistence of foragers in returning to the same food source (Figure S1a). However, the optimal proportions of scouts are smaller than those reported in the main text, when recruits do not recruit (Figure S1b) and the amounts of resources collected are larger (Figure S1c). Furthermore, adding recruitment by recruits does not change the opposite relationship between the optimal proportion of scouts and the persistence of scouts vs. the persistence of recruits that we report in the main text (Figure S1d,e). When the persistence of recruits is fixed, the optimal proportion of scouts grows almost linearly with the persistence of scouts (Figure S1f); when the persistence of scouts is fixed, the optimal proportion of scouts decays with the persistence of recruits and plateaus between 20% and 40% (Figure S1g).

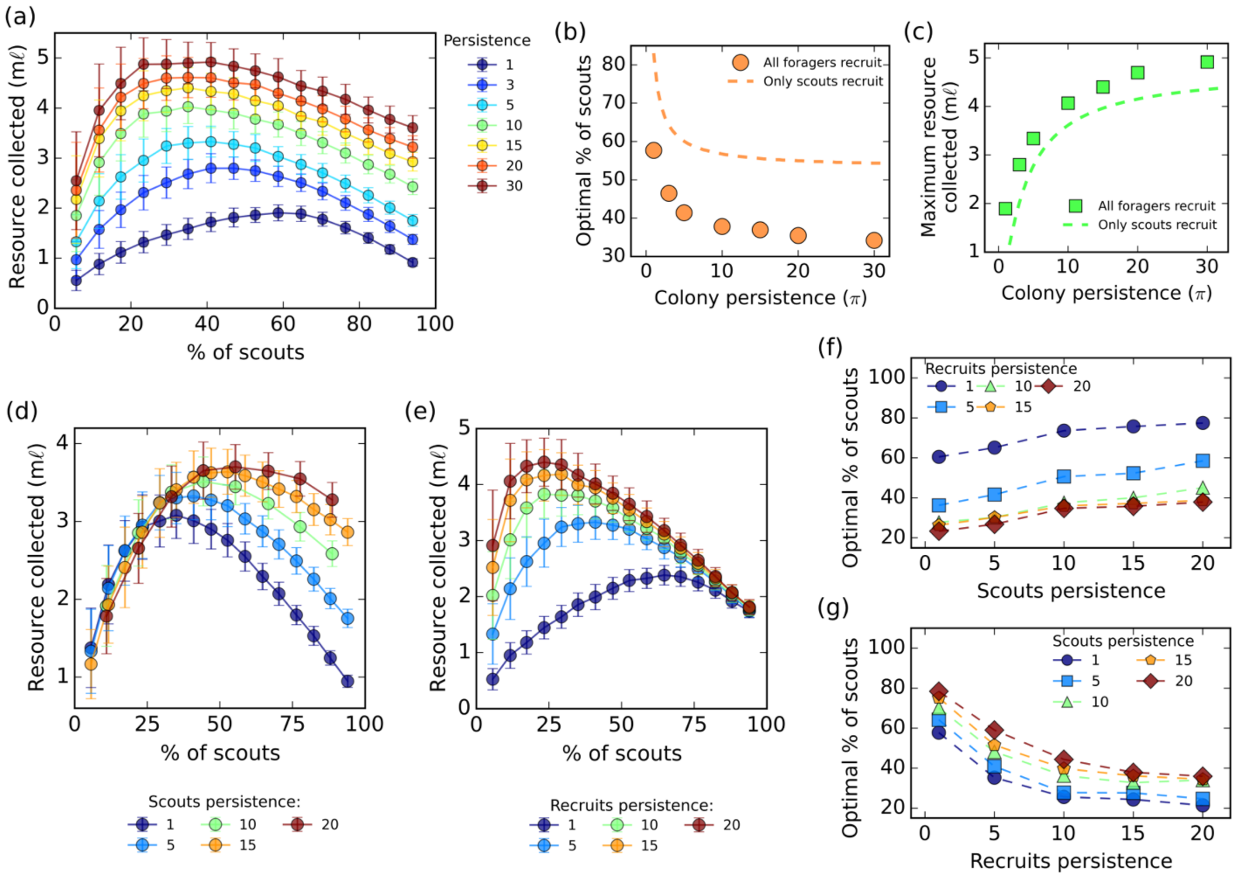

Figure S1. Effects of including recruitment by recruits in our model. The relationship between persistence and the optimal proportion of scouts does not change. **(a)** Total amount of resource collected throughout the entire simulation as a function of the proportion of scouts in the colony for different values of persistence of all foragers (π). Bars represent the standard deviation across all simulation runs. Compare with Figure 3(c). **(b)** The optimal proportion of scouts plateaus near 35% as π increases. Dashed line represents the results from Figure 3(d). **(c)** Maximum amount of resources collected scales sublinearly with π. Dashed line represents the results from Figure 3(e). **(d,e)** Total amount of resources collected by a colony as a function of the proportion of scouts when (d) the persistence of scouts is set to $\pi^s = 5$ for the following values of persistence for recruits: π^r =1,5,10,15,20; and (e) the persistence of recruits is set to $\pi^r = 5$ for the following values of persistence of scouts: π^s =1,5,10,15,20. Compare with Figures 4(a,b). **(f)** Optimal proportion of scouts as a function of recruit persistence for different values of fixed scout persistence π^s. Compare with Figure 4(c). **(g)** Optimal proportion of scouts as a function of scout persistence for different values of fixed scout persistence π^r. Compare with Figure 4(d).

Boundary conditions

Making bees that reach the boundary of the simulated foraging area fly back to the hive in a straight line did not change our findings compared with returning them to the hive instantaneously. Including this trip back to the hive created a time delay during which scouts that go beyond the simulated area cannot recruit. This time delay is a function of the distance of foragers from the hive and their flight velocity. Assuming that the average velocity remains the same throughout the simulation, then this time delay has a similar effect as reducing the recruitment rate (compare Figures S9a with S1 and S6). The relationship between the optimal proportion of scouts and colony persistence remains the same as in all the various iterations of our model (Figure S9b). Furthermore, the total amount of collected resources will increase monotonically with this delay (Figure S9c). For a small distance such as 50m, no difference was observed when adding a return trip, compared with returning bees to the hives instantaneously.

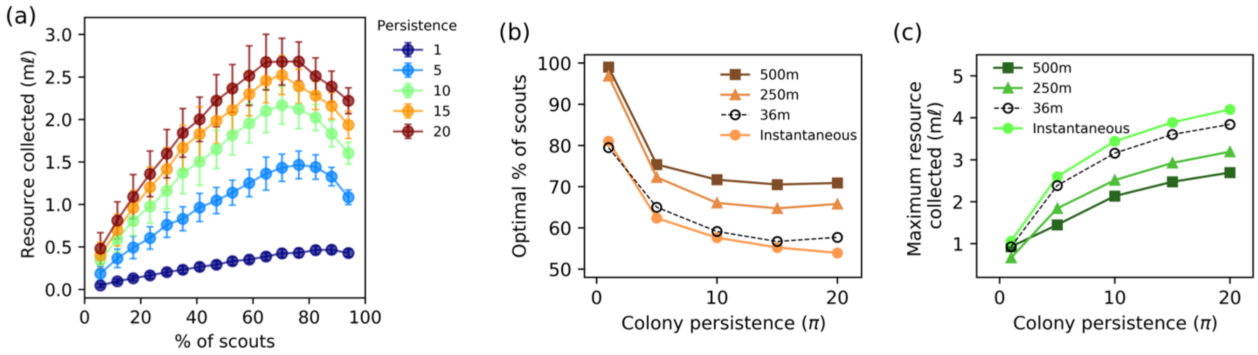

Figure S2. The effect of the distance that a simulated bee flies before returning to the hive to start foraging again. (a) Total amount of resource collected as a function of the proportion of scouts in the colony for different values of persistence. Distance was set to 500m. Bars represent the standard deviation across all simulation runs. **(b)** The optimal proportion of scouts slowly increases with the total distance explored by the simulated bees, saturating when this distance approaches 500m. Orange circles are the results shown in the paper (Figure 3d). **(c)** The maximum amount of resources collected by the colony decreases with the total distance explored by the simulated bees. Green circles are the results shown in the paper (Figure 3e).

Experiments with feeders at different distances

To fit the average velocity of the simulated honey bees and the time that they spend in the hive recruiting other foragers, we empirically determined the time interval between consecutive visits when feeders were positioned at four different distances from the hive (3, 5, 10 and 15m). The observed inter-visit interval $< t >$ was linearly related to the distance d between hive and feeder (R = 95%, Figure S3a): $< t > = \alpha d + \beta$, with $\alpha = 0.28 \pm 0.05$ ($p < 0.01$) and $\beta = 2.3 \pm 0.3$ ($p < 0.01$). We used these empirical values to set the parameters of our model. Persistence was not affected by distance from the hive (Figure S3b).

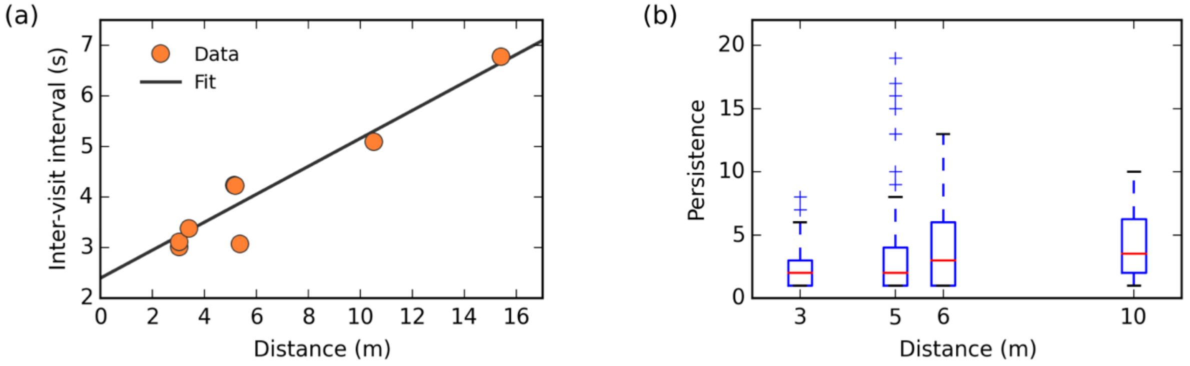

Figure S3. Results from experiments with the feeder located at different distances. (a) Average time between two consecutive visits to the feeder grows linearly with the distance. **(b)** Persistence values at different distances.

Number of resource patches

Changing the number of resource patches did not affect our main findings. For each persistence, there was always a group composition that maximized the amount of resources collected (see Figure S3a for one patch). Models with fewer patches had a greater variance in the total amount of resources collected (vertical bars in Figure S3a). Although the optimal composition decreased with persistence, this decrease was sharper for environments with more patches (Figure S3b). Finally, as expected, environments with more patches increased the total amount of resources collected (Figure S3c). The maximum amount of resources collected can be approximated by a saturating exponential model, $A_0 - A_1 e^{\alpha\pi}$, with π being persistence (see lines in Figure S3c).

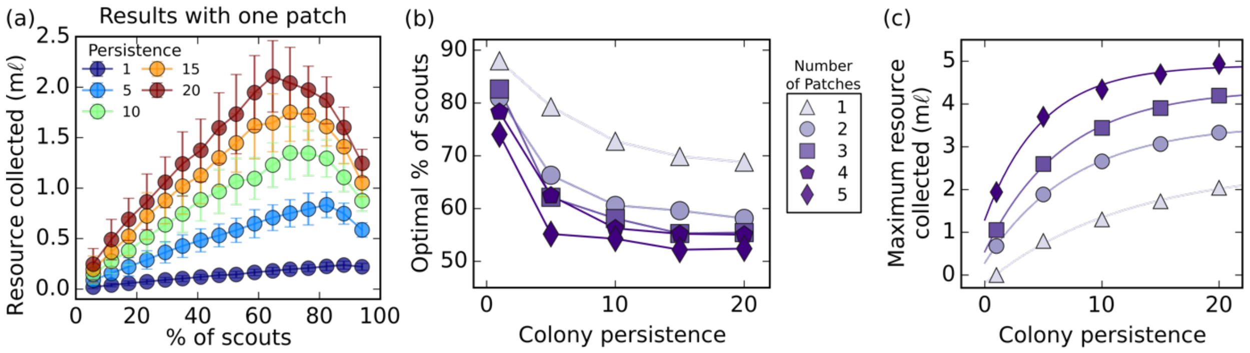

Figure S4. Changing the number of resource patches in the environment did not affect the relationship between optimal colony composition and persistence. (a) Amount of resources collected as a function of the percent of scouts when all resources were in a single patch. **(b)** With more patches, the optimal proportion of scouts decreased faster with persistence. **(c)** Maximum amount of resources collected always increased with the number of patches.

Comparing predictions between the System Dynamics and Agent-Based Models

The curvature of the dependence between the amount of resources collected and the proportion of scouts slightly differs between the Agent-Based and the Systems Dynamics models (Figure S4). These differences emerge from the fact that the only non-linearity in our Systems Dynamics model is the recruitment term $-S \times R$ (Equation 5b), resulting in curves that are parabolas, as predicted by Equation 10. However, the Agent-Based Model is comprised by many non-linear interactions and processes that are difficult to express analytically. For instance, recruitment was modeled as a stochastic contact process that lasts for a variable period of time; the spatially-explicit distribution of resources slightly deviates from a uniform distribution, which in the systems dynamics model is assumed completely uniform by the fixed the rate γ_d at which scouts find new resources; and the flight dynamics in the Agent-Based model is piece-wise continuous, with frequent changes to its functional form (i.e., flight dynamics change from a drifting random walk to movement in a straight line and then to stopping at the hive). Each of these processes change the shape and curvature of the simulated relationship between the amount of resources collected and the proportion of scouts.

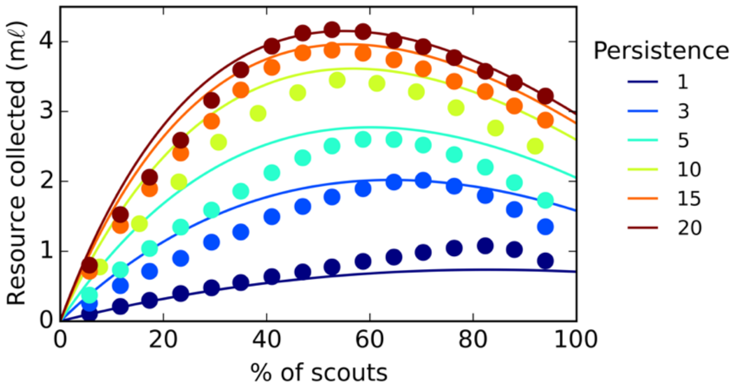

Figure S5. Comparing the predicted curves for food collection in relation to the proportion of scouts between the Systems Dynamics model (Equations 5a, 5b and 6, lines) and the Agent-Based model (points).

Increasing the rate of recruitment

Increasing the rate of recruitment decreased the proportion of scouts that resulted in the maximum amount of food collected (Figure S5a), regardless whether recruitment takes place only during the first return trip or during all return trips. Recruitment rate was measured as the average number, K, of bees that were recruited by each waggle dance. When K was greater than 40, the optimal proportion of scouts became very small (below 10% for all persistences). Although the values of the optimal proportion of scouts became smaller, the relationship with colony persistence remained unchanged compared to what we report in the main text. At K=25 the optimal proportion of scouts ranged between 20% and 40% (Figure S5a), in agreement with the estimated percentage of scouts in honeybee colonies [2]. The relationship between the optimal proportion of scouts and the maximum amount of resources collected as a function of the colony persistence remained the same as reported in the main text (Figures S5b,c).

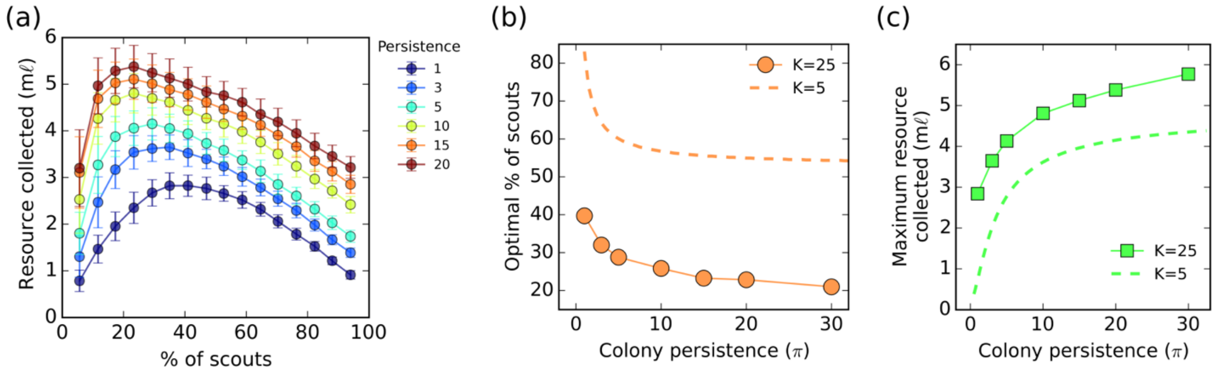

Figure S6. Effects of increasing the rate of recruitment by scouts. Recruitment rate is defined as the number, K, of bees recruited by each waggle dance. We compare K = 5 to K=25, which we used in the main text. **(a)** Total amount of resource collected as a function of the proportion of scouts in the colony for different values of persistence. Bars represent the standard deviation across all simulation runs. **(b)** The optimal proportion of scouts ranges from 24% to 40%, considerably smaller than those shown in the main text (dashed line corresponds to Figure 3d). **(c)** Maximum amount of resources collected scales sublinearly with π. Dashed line represents the results from Figure 3(e).

Colony size

Regardless of colony size, the proportion of scouts required for collecting the maximum amount of resources (optimal composition) decreased with persistence (Figure S6a). In the main text, we show results from simulations with 300 scouts. The exponential decays for all colony sizes were very close to 40 ± 5 (% scouts/persistence). Although colonies of different sizes showed slightly different optimal compositions, these differences saturated above 900 bees (Figure S6b). Regardless, larger colonies always collected more resources than small ones when given the same amount of time to forage (Figure S6c). For all colony sizes, the maximum amount of resources collected can be approximated by a saturating exponential model, $A_0(1 - e^{\alpha\pi})$, with π being persistence (see lines in Figure S6c). Including recruitment by recruits did not change our findings (Figure S8).

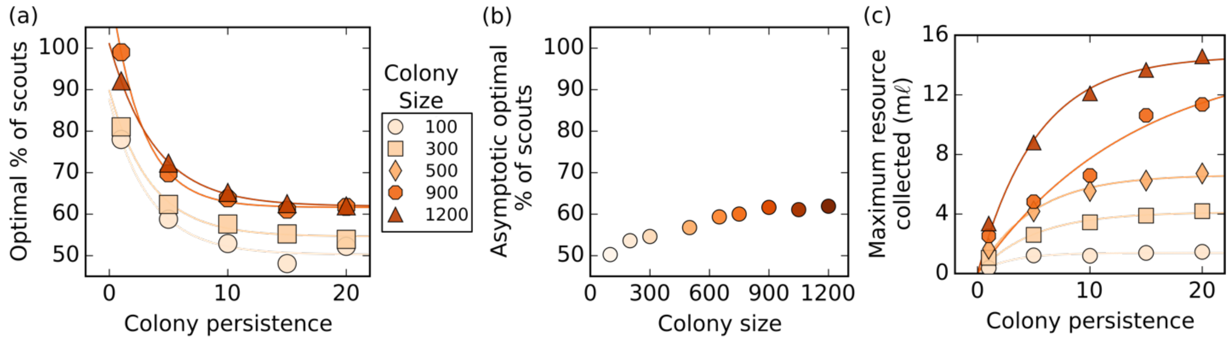

Figure S7. The size of a colony influences the total amount of resources collected during the simulated foraging, but has little impact on the colony composition that optimizes the collection of resources. (a) For all colony sizes tested, the optimal percent of scouts decreased with persistence. Lines are the fit of an exponential decay to the simulation results. **(b)** Optimal percent of scouts at the asymptote of the exponential decay changes about 10% with colony size and then stablizes. **(c)** Maximum amount of resources collected increased with colony size. Lines are the fit of an exponential model to the simulaiton results.

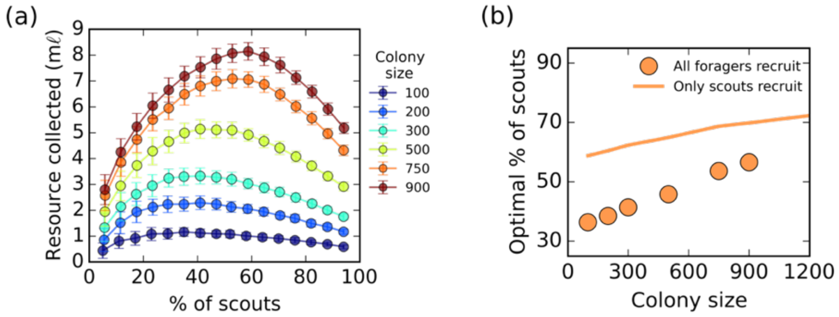

Figure S8. Relationship between recruitment by recruits and colony size. (a) Total amount of resource collected as a function of the proportion of scouts in the colony for different colony sizes. Bars represent the standard deviation across all simulation runs. **(b)** The optimal proportion of scouts grows linearly with colony size. The rate at which the proportion of scouts change is faster than that of the results without recruitment by recruits. Persistence for all foragers in both panels was set to $\pi = 5$.

Increasing flight precision in response to finding a resource

Removing the increase in flight precision after detecting a new resource did not change our main findings. The increase in flight precision that we included in our model reflects the communication of information about distance and direction between scouts and recruits [1]. The proportion of scouts that led to an optimal amount of resources collected decreased with persistence in the presence and absence of increasing flight precision (Figure S7a). However, the total amount of resources that a colony collected when there was an increase in flight precision after finding a resource was substantially larger than without this increase (Figure S7b). In both cases, the maximum amount of resources collected can be approximated by a saturating exponential model, $A_0(1-e^{\alpha\pi})$, with π being persistence, and A_0 and α fitted using the simulated results (see lines in Figure S7b).

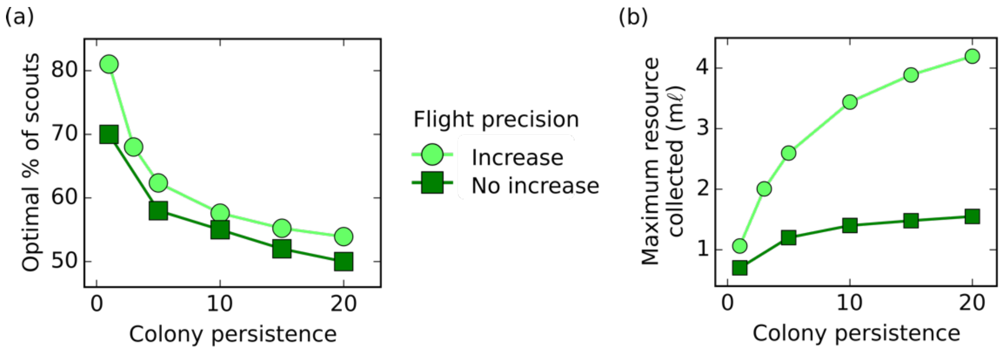

Figure S9. The effect of increasing flight precision after finding a new resource. (a) The relationship between optimal colony composition and behavioral persistence was not affected by increasing (light green) flight precision after finding a resource. **(b)** Total amount of resources collected with an increase in flight precision (light green) was on average 2.5 larger than without this increase (dark green). Lines represent the exponential fit to the simulated data.

References

1. Frisch, K. von 1967 *The Dance Language and Orientation of Bees*. Cambridge: Harvard University Press.
2. Seeley, T. D. 1983 Division of labor between scouts and recruits in honeybee foraging. *Behav. Ecol. Sociobiol.* **12**, 253–259. (doi:10.1007/BF00290778)
